# TCF7L2 positively regulates aerobic glycolysis via the EGLN2/HIF-1α axis and indicates prognosis in pancreatic cancer

**DOI:** 10.1038/s41419-018-0367-6

**Published:** 2018-02-23

**Authors:** Jinfeng Xiang, Qiangsheng Hu, Yi Qin, Shunrong Ji, Wenyan Xu, Wensheng Liu, Si Shi, Chen Liang, Jiang Liu, Qingcai Meng, Dingkong Liang, Quanxing Ni, Jin Xu, Bo Zhang, Xianjun Yu

**Affiliations:** 1 Department of Pancreatic Surgery, Fudan University Shanghai Cancer Center, 200032 Shanghai, China; 2 Department of Oncology, Shanghai Medical College, Fudan University, 200032 Shanghai, China; 3 Pancreatic Cancer Institute, Fudan University, 200032 Shanghai, China; 4 Shanghai Pancreatic Cancer Institute, 200032 Shanghai, China

## Abstract

Patients with pancreatic ductal adenocarcinoma have much worse prognoses, and much effort has been directed toward understanding the molecular biological aspects of this disease. Accumulated evidence suggests that constitutive activation of the Wnt/β-catenin signalling contributes to the oncogenesis and progression of pancreatic cancer. Transcription factor 7-like2/transcription factor 4 (TCF7L2/TCF4), a β-catenin transcriptional partner, plays a vital role in the Wnt/β-catenin signalling pathway. In the present study, we investigated the clinicopathological significance of TCF7L2 in pancreatic cancer. Our results demonstrated that patients with higher TCF7L2 expression had worse prognosis. Our in vitro studies demonstrated that TCF7L2 positively regulated aerobic glycolysis by suppressing Egl-9 family hypoxia inducible factor 2 (EGLN2), leading to upregulation of hypoxia inducible factor 1 alpha subunit (HIF-1α). The impact of TCF7L2 on aerobic glycolysis was further confirmed in vivo by assessing ^18^FDG uptake in pancreatic cancer patients and in a subcutaneous xenograft mouse model. In summary, we identified novel predictive markers for prognosis and suggest a previously unrecognized role for TCF7L2 in control of aerobic glycolysis in pancreatic cancer.

## Introduction

Pancreatic ductal adenocarcinoma (PDAC) is one of the most aggressive diseases, with remarkably highly lethality rates. Surgical techniques have made great progress over recent decades; however, only 15–20% of patients with pancreatic cancer meet the conditions for receiving operations, and the overall 5-year survival rate of patients with pancreatic cancer has remained at approximately 6%^1,2^. A potential reason is the late diagnosis of pancreatic cancer in clinical practice, and the most plausible explanation for this situation is that pancreatic cancer tends to invade nearby tissues or metastasize to distant organs through blood or lymph vessels, which makes it unresectable and uncontrollable^3^. Thus, there is an urgent need for a better understanding of the molecular mechanisms that underlie the tumourigenesis and progression of pancreatic cancer^4^. Similar to other types of malignant cancer, pancreatic cancer has a pattern of high proliferation, invasion, and metastasis activity^5^. Large-scale changes in cellular metabolism occur along with these cancerous processes in order to satisfy high demands for material and energy^6^. These problems are much more challenging for pancreatic cancer, since its abundant fibrotic stroma create a unique nutrient-deficient microenvironment^7,8^. Thus, pancreatic cancer cells need to reprogram metabolic pathways to accommodate this hostile microenvironment with scarce nutrients and oxygen^9^. The hypoxic adaption mediated by the master transcriptional regulator, HIF-1α, is the best characterized metabolic alteration, which mediates the survival of tumour cells to survive by aerobic glycolysis reprogramming^10^. This process is known as the “Warburg effect”, in which glucose is metabolized through glycolysis to generate ATP and basic raw materials for biosynthesis^11,12^. Therefore, HIF-1α-mediated metabolic reprogramming creates a specific matrix with suitable acid-base characteristics and promotes angiogenesis for invasion and metastasis, which correlates closely with resistance to chemotherapy and radiotherapy and may also serve as a treatment target^13^.

Pancreatic cancer is characterized by diverse mutations such as RAS, Smad4, p53, and inappropriate activation of crucial embryonic signalling pathways including Notch, Hedgehog, and Wnt signalling, which are essential for embryonic development and tissue homeostasis^2,14^. Of particular interest is that a considerable portion of these mutations and pathways not only contribute to the ability of tumour cells to proliferate and differentiate but also alter cell metabolic plasticity to support urgent requirements for oxygen and nutrients^15^. To date, a massive interdisciplinary effort has illuminated the Wnt signalling transduction cascade, which is involved in various aspects of embryonic development and self-renewal homeostasis in adult tissues. Calling for special attention is the fact that some germline mutations of the Wnt pathway could cause several hereditary diseases. Moreover, somatic mutations are associated with some types of gastrointestinal cancer^16^.

In normal development, activation of the canonical Wnt/β-catenin cascade depends on secreted Wnt ligands that activate the Frizzled(Fz)/LRP co-receptor complex. Frizzled interacts with Dishevelled (Dsh) to activate the GSK3β kinase and casein kinase I-γ (CK1γ), which induce the phosphorylation of Dsh and LRP. Thus, Wnt signalling controls the recruitment of Axin away from the destruction complex, which results in the stabilization of β-catenin. Finally, in the nucleus, β-catenin displaces Groucho from TCF4/LEF to promote the transcription of Wnt target genes^17^. As for PDAC, aberrantly persistent accumulation of β-catenin is a common phenomenon, which results in the activation of target genes along with TCF4/LEF co-factors^15^. Functional evidence has revealed that the Wnt/β-catenin/TCF cascade plays a critical role in oncogenesis and metastasis of pancreatic cancer^18^. However, the contribution of the Wnt/β-catenin pathway and related regulatory factors in pancreatic cancer cell metabolism is not clearly understood. In the present study, we discovered that the expression of TCF7L2 was positively correlated with aerobic glycolysis and was coincident with the analytic results of PET/CT from human and small laboratory animals. We further found that TCF7L2 might affect aerobic glycolysis by regulating the EGLN2/HIF-1α axis. Furthermore, we demonstrated that TCF7L2, EGLN2, and glycolysis markers positively correlated with one another, through analysis of the Cancer Genome Atlas (TCGA) dataset and by immunohistochemical staining (IHC) staining in pancreatic cancer tissue samples from Fudan University Shanghai Cancer Center (FUSCC). Our present study sheds light on novel predictive and treatment targets for pancreatic cancer.

## Results

### Overexpression of TCF7L2 indicates a poor prognosis for patients with pancreatic cancer

**Fig. 1:**
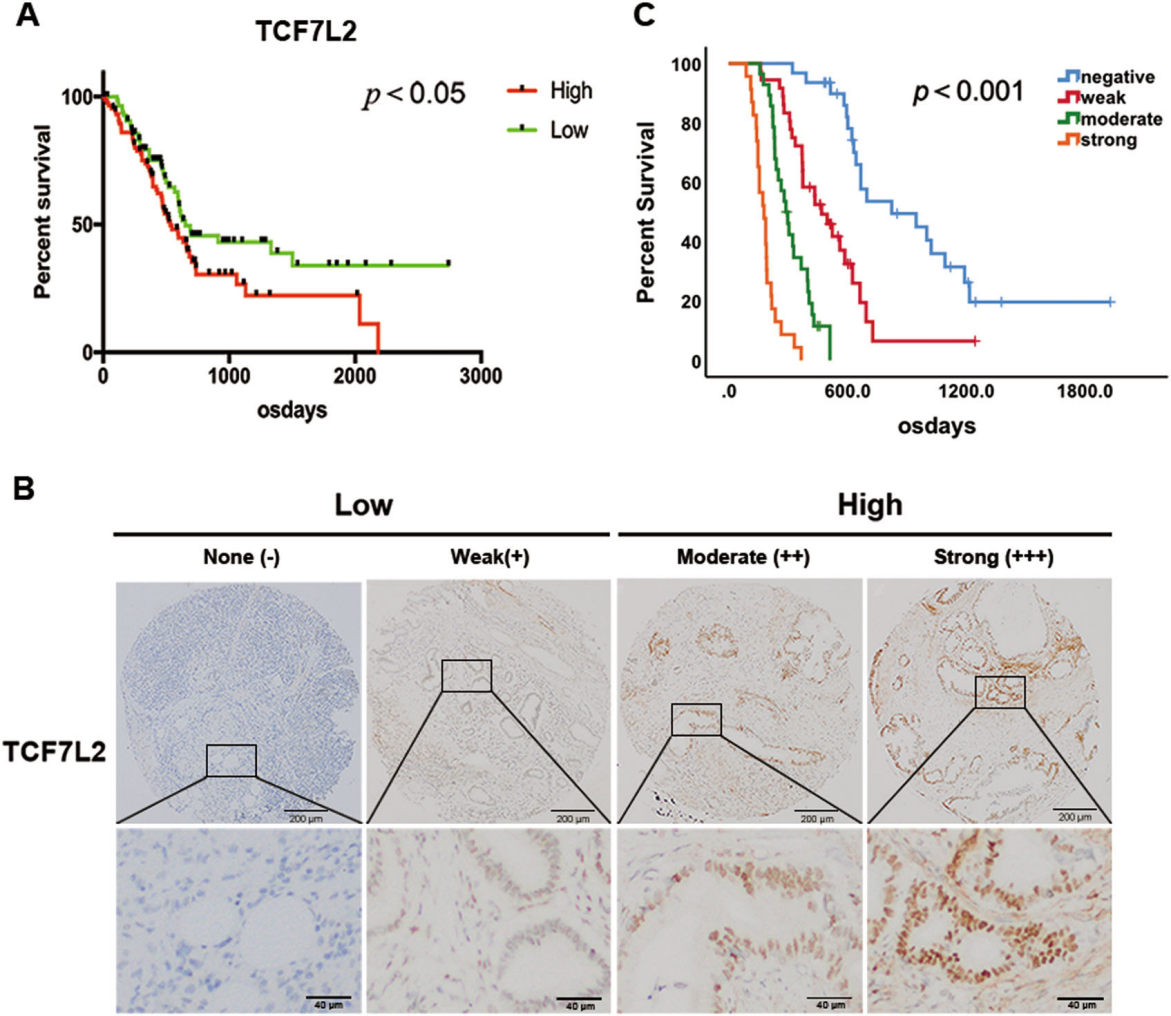
TCF7L2 expression is significantly related to the overall survival of patients with pancreatic cancer. **a** Survival analysis of TCF7L2 in TCGA dataset. High TCF7L2 expression indicated worse prognosis. *P* < 0.05 vs. low group. **c** Survival analysis of TCF7L2 in FUSCC dataset. *P* < 0.001 vs. negative group. **b** IHC scoring of TCF7L2 expression in tissue samples from patients with PDAC (magnification scale bar, 200 and 40 μm)

To explore the impact of TCF7L2 on pancreatic cancer patient prognosis, we examined the expression of TCF7L2 in TCGA pancreatic cancer patients. As shown, higher levels of TCF7L2 expression indicated a worse prognosis (Fig. 1a). To further validate this finding, we examined TCF7L2 expression by tissue microarray of 118 IHC-stained pancreatic cancer tissues from FUSCC. Moreover, we performed a detailed evaluation of TCF7L2 staining based on IHC scoring. The standard for IHC scoring of TCF7L2 is shown in Fig. 1c. We performed a Kaplan–Meier analysis stratified by TCF7L2 expression, and we further detected and analyzed the TCF7L2 expression status and prognosis in patients with pancreatic cancer from FUSCC. These results demonstrated that TCF7L2 expression was significantly related to the overall survival (OS) of pancreatic cancer patients (Fig. 1b). Moreover, the OS for pancreatic cancer patients with higher TCF7L2 expression was significantly shorter than for patients with low TCF7L2 expression (*P* < 0.001, median survival time: 33.6 months vs. 17.1 months vs. 10.5 months vs. 6.2 months). Moreover, we analyzed the correlation between the TCF7L2 expression and clinicopathological features in clinical samples of PDAC from the FUSCC dataset. The results showed that high TCF7L2 expression was closely related to a low degree of tumour differentiation (Table 1, *P* = 0.029) and to the status of lymph node metastasis (Table 1, *P* = 0.042). Thus, we hypothesized that TCF7L2 may play an important role in the incidence or development of pancreatic cancer and could be closely related with prognosis in pancreatic cancer.

**Table 1:** Clinicopathological features and correlation of TCF7L2 expression in patients with PDAC from FUSCC

		TCF7L2^Low^	TCF7L2^High^	Spearman correlation	*P*-value
Characteristics	No.	Score (−/+) (*n* = 67)	score (++/+++) (*n* = 51)		
Age (years)				0.061	0.514
<60	55	33	22		
≥60	63	34	29		
Gendar				−0.056	0.547
Male	61	33	28		
Female	57	34	23		
Tumor size (cm)				0.095	0.306
≤3.0	55	34	21		
>3.0	63	33	30		
Tumor differentiation				−0.201	0.029
Well	22	16	7		
Moderate	58	36	22		
Poor	38	15	7		
Lymph node status (stage)				0.187	0.042
N0	64	42	22		
N1	48	22	26		
N2	6	3	3		
Vessel infiltration				0.037	0.689
Negative	81	47	34		
Positive	37	20	17		
Nerve infiltration				−0.041	0.657
Negative	21	11	10		
Positive	97	56	41		

TCF7L2^Low^: negative/weak TCF7L2 expression; TCF7L2^High^: moderate/strong TCF7L2 expression; lymph node status (stage) was defined by the AJCC 8th edition (N0: node negative, N1: 1–3 nodes positive for metastatic disease, N2: more than four nodes positive for metastatic disease); *P-*values were derived with Spearman rank correlation coefficient test; all statistical tests are two-sided

### TCF7L2 positively regulates the proliferation of pancreatic cancer

**Fig. 2:**
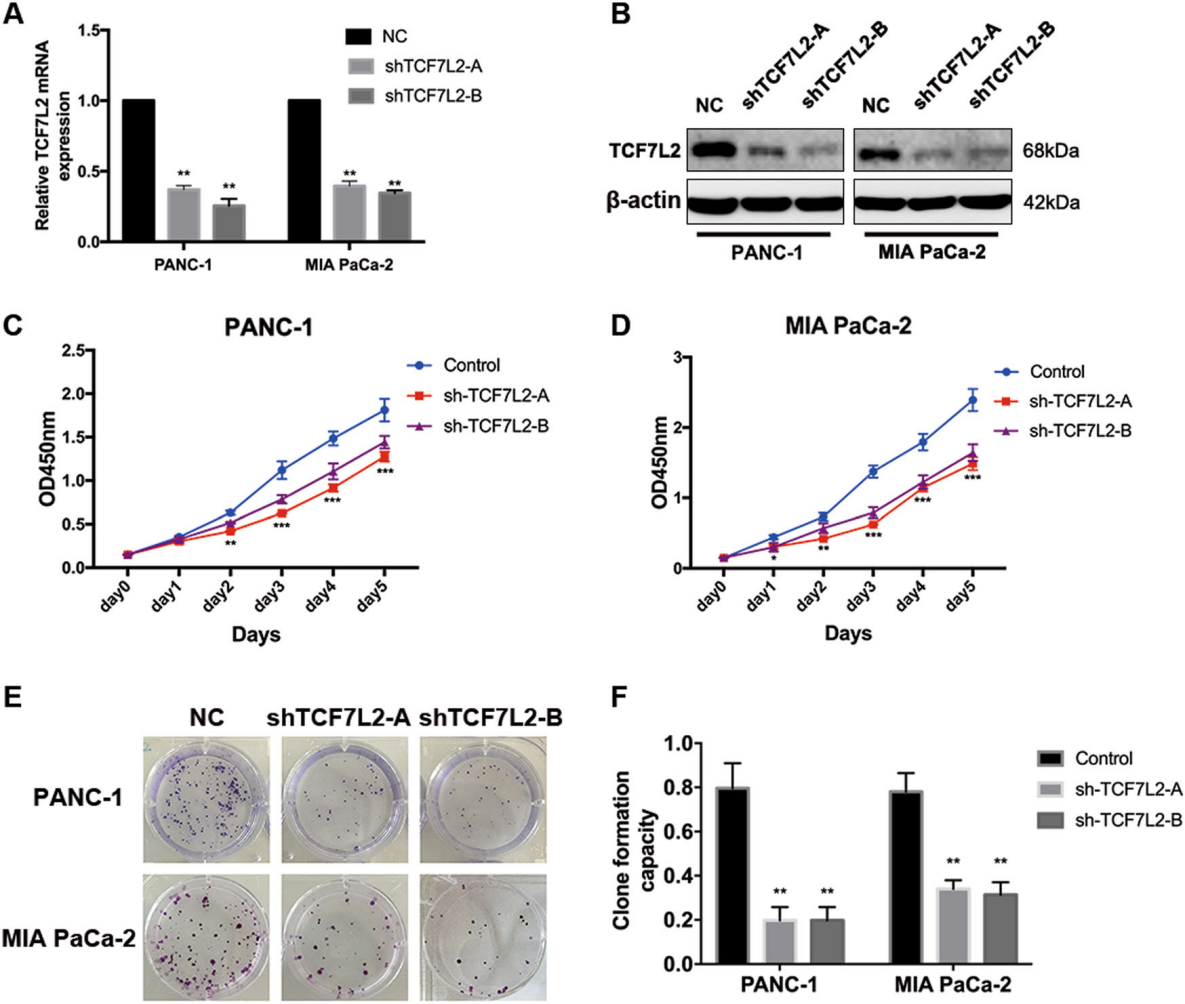
TCF7L2 positively regulates the proliferation of pancreatic cancer. **a** Quantitative RT-PCR analysis of TCF7L2 knockdown efficiency in PANC-1 and MIA PaCa-2 cell lines that stably express shRNA oligoes against TCF7L2. **P < 0.01 vs. control group. **b** Western blot analysis further confirmed the silencing efficacy. **c**, **d** CCK-8 proliferation assay indicated that TCF7L2 silencing decreased the viability of PANC-1 and MIA PaCa-2 cells. **e**, **f** TCF7L2 inhibited the clone formation capacity of PANC-1 and MIA PaCa-2 cell lines. **P < 0.01 vs. control group

To further evaluate the function of TCF7L2 in pancreatic cancer viability and proliferation, we first generated two lentiviral particles targeting TCF7L2, termed pLKO.1-shTCF7L2-A and pLKO.1-TCF7L2-B, to mediate silencing of TCF7L2 in PANC-1 and MIA PaCa-2 cells. We next infected PANC-1 and MIA PaCa-2 cells with lentivirus, followed by puromycin screening to obtain cell lines stably expressing shRNAs against TCF7L2. The efficiency of knockdown was validated by real-time PCR and western blotting (Fig. 2a, b). Then, we performed CCK-8 proliferation assays to validate the influence of TCF7L2 on pancreatic cancer viability. As shown, knockdown of TCF7L2 significantly reduced cancer cell viability of PANC-1 and MIA PaCa-2 cells (Fig. 2c, d). Moreover, we also performed a clone formation assay. These results revealed that knockdown of TCF7L2 significantly inhibited the clone formation capacity of PANC-1 and MiaPaCa-2 cells, supporting a role for TCF7L2 in pancreatic cancer cell proliferation (Fig. 2e, f).

### TCF7L2 positively regulates aerobic glycolysis and ^18^F-FDG uptake in pancreatic cancer

**Fig. 3:**
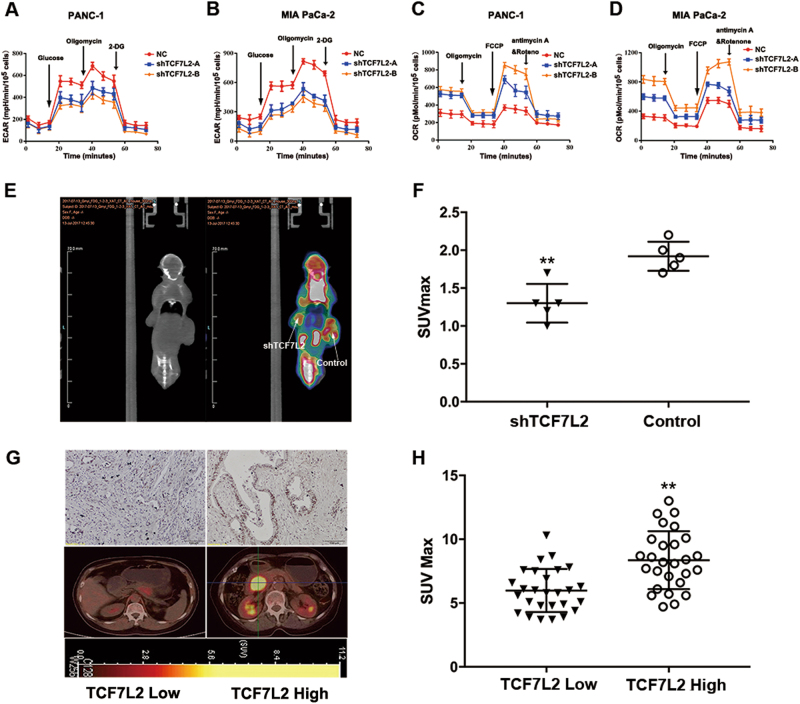
TCF7L2 is positively related with aerobic glycolysis and ^18^F-FDG uptake in pancreatic cancer. **a**, **b** TCF7L2 knockdown decreased glycolysis capacity, which could be shown by the ECAR test in PANC-1 and MIA PaCa-2 cells. **c**, **d** OCR significantly increased in TCF7L2-silenced PANC-1 and MIA PaCa-2 cells, indicating TCF7L2 functions as a positive regulator of mitochondrial respiration. **e**, **f** TCF7L2 expression positively correlated with ^18^F-FDG uptake in the in vivo xenograft model. **P < 0.01 vs. control group. **g**, **h** Higher TCF7L2 expression was always accompanied by higher SUV_max_ values in patients with pancreatic cancer. **P < 0.01 vs. TCF7L2 low expression group

The roles of TCF7L2 in tumourigenesis and development have received much attention in recent years. Our above-mentioned observations also indicate that TCF7L2 plays crucial roles in proliferation in pancreatic cancer. However, its contribution to aerobic glycolysis, a recently appreciated characteristic of cancer, has rarely been studied. On the other hand, many previous studies have shown that the SUV_max_ value in PET/CT reports, which reflects the glycolytic capacity of cancer cells, was closely related with prognosis in several tumour types, including pancreatic cancer. To explore the potential relationship between TCF7L2 and glucose metabolism in pancreatic cancer, we used Seahorse XF Extracellular Flux Analyzers to analyze the effect of TCF7L2 on aerobic glycolysis. The glycolytic process (extracellular acidification rate, ECAR) significantly decreased in TCF7L2-silenced PANC-1 and MIA PaCa-2 cells, which implied that TCF7L2 has a positive effect on the glycolysis rate in pancreatic cancer cells (Fig. 3a, b). Detection of mitochondrial respiration (oxygen consumption rate, OCR) was also carried out. The results showed that OCR increased significantly in PANC-1 and MIA PaCa-2 cells with TCF7L2 knockdown, which confirmed the hypothesis that TCF7L2 is closely involved in glycolysis (Fig. 3c, d). To further validate the in vitro function of TCF7L2 in glucose metabolism, we subcutaneously injected nude mice with TCF7L2-silenced PANC-1 cells. Then, we used a small animal imaging system to simultaneously detect tumour size and tumour-associated glucose uptake. Silencing TCF7L2 expression inhibited tumour growth and significantly attenuated ^18^F-FDG uptake in this in vivo xenograft model (Fig. 3e, f). Furthermore, we detected the expression status of TCF7L2 by IHC staining and analyzed its correlation with ^18^F-FDG uptake in pancreatic cancer patients. The results demonstrated that higher TCF7L2 expression was always accompanied by a higher SUV_max_ value (Fig. 3g, h). Based on the above observations, we hypothesized that TCF7L2 was a positive regulator of aerobic glycolysis in vitro in cancer cell lines and in vivo in pancreatic cancer patients.

### TCF7L2 positively regulates HIF-1α stability and relevant glycolysis genes in pancreatic cancer

**Fig. 4:**
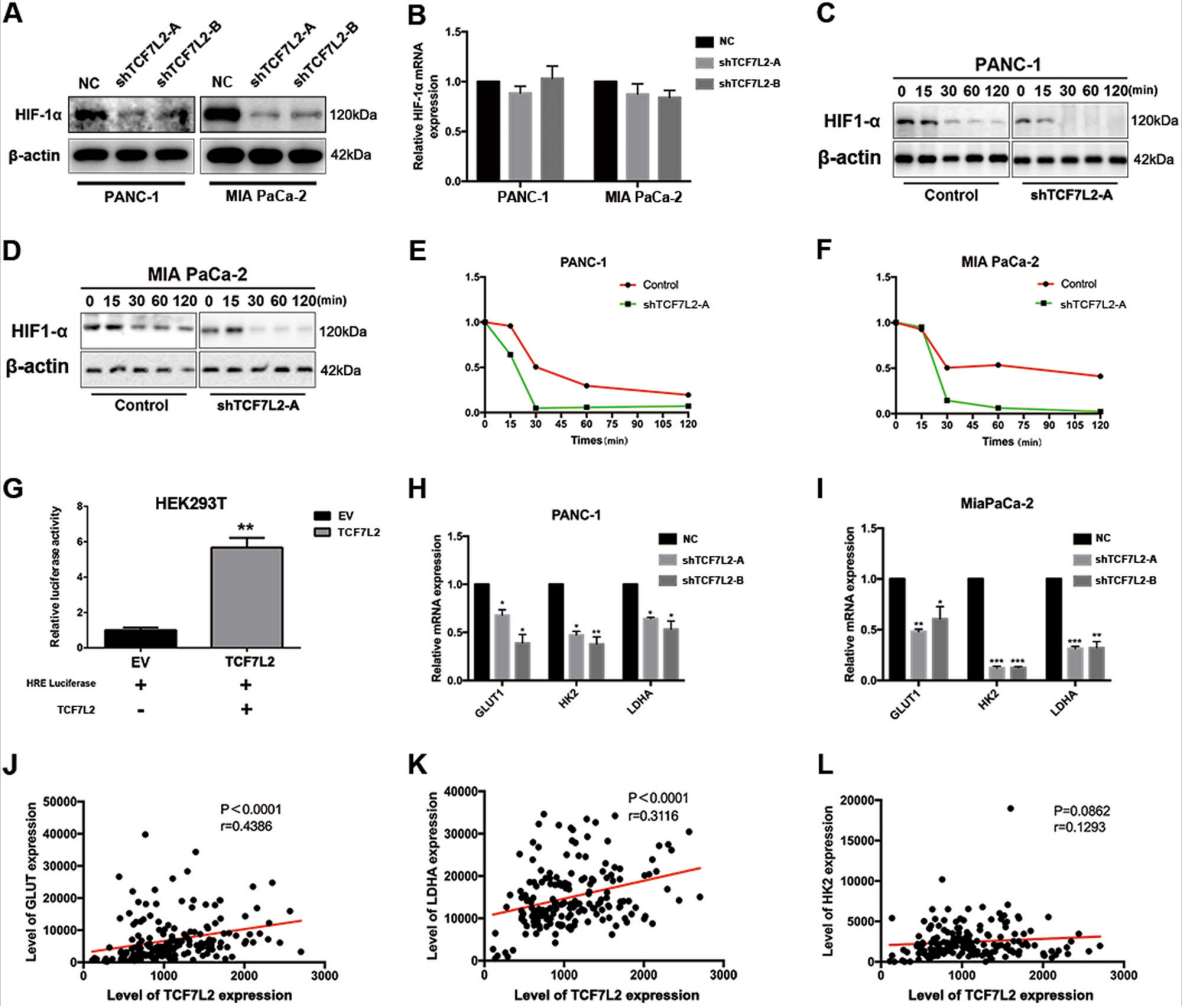
TCF7L2 is positively correlated with HIF-1α stability and relevant glycolysis genes (GLUT1, HK2, LDHA) in pancreatic cancer. **a** TCF7L2 silencing decreased the protein level of HIF-1α. **b** There was no significant change in the expression of HIF-1α at the transcriptional level in TCF7L2-silenced PANC-1 and MIA PaCa-2 cells. **c**–**f** Kinetics of HIF-1α protein degradation. HIF-1α protein degraded faster in shTCF7L2 cells than in controls in PANC-1 (**c**, **e**) and MIA PaCa-2 cells (**d**, **f**). **g** TCF7L2 could upregulate HRE-luciferase activity in HEK293T cells. **P < 0.01. **h**, **i** TCF7L2 silencing inhibited the expression of HIF-1α-targeted glycolytic genes, including GLUT1, HK2, and LDHA in PANC-1 and MIA PaCa-2 cells. *P < 0.05, **P < 0.01, ***P < 0.001 vs. control group. **j**, **k** TCF7L2 positively and significantly correlated with GLUT1 and LDHA expression in pancreatic cancer patients. **l** TCF7L2 positively correlated with HK2 expression, but this did not reach statistical significance

Pancreatic cancer always exhibits an increased accumulation of stromal tissue with the features of desmoplasia and hypoxia, which lead to the stabilization of hypoxia inducible factor-1a (HIF-1α), the master regulator of glucose metabolism. Previous studies have revealed overexpression of HIF-1a and relevant downstream glucose metabolism genes in pancreatic cancer. To explore the specific mechanisms underlying TCF7L2-mediated glycolysis regulation, we validated the HIF-1α expression levels by western blot and quantitative real-time PCR in TCF7L2 knockdown PANC-1 and MIA PaCa-2 cells. As shown, HIF-1α protein level significantly decreased in TCF7L2-silenced PANC-1 and MIA PaCa-2 cells (Fig. 4a). However, there was no significant change in the expression of HIF-1α at the transcriptional level (Fig. 4b). Thus, we hypothesized that TCF7L2 might regulate HIF-1α protein stability. To prove this, we transfected PANC-1 and MIA PaCa-2 cells with a HIF-1α expressing plasmid. To measure the kinetics of HIF-1α protein degradation, we treated shTCF7L2 and control cells with CoCl_2_ to stabilize the HIF-1α protein. Then, we added cycloheximide (CHX) to arrest protein synthesis. The degradation rates of the existing HIF-1α protein were observed by measuring HIF-1α and actin levels in cell lysates harvested at 0, 15, 30, 60, and 120 min after CHX addition. We observed that HIF-1α protein degraded faster in shTCF7L2 cells than in controls, which was confirmed by densitometry analysis of immunoblotting results (Fig. 4c–f). Next, we assessed the impact of TCF7L2 on HIF-1α transcriptional activity, demonstrated by HRE-luciferase assay (Fig. 4g). As shown, TCF7L2 could upregulate HRE-luciferase activity in HEK293T cells. HIF-1α regulated aerobic glycolysis by targeting glycolysis genes, such as GLUT1, HK2, and LDHA. We observed that in TCF7L2-silenced PANC-1 and MIA PaCa-2 cells, the mRNA expression of GLUT1, HK2, and LDHA decreased (Fig. 4h, i). Finally, we validated these observation in pancreatic cancer patients. As shown, TCF7L2 significantly positively correlated with GLUT1 and LDHA (Fig. 4j, k). Although TCF7L2 positively correlated with HK2 expression, this correlation was not statistically significant (Fig. 4l).

### EGLN2 negatively correlates with TCF7L2 expression and indicated prognosis in pancreatic cancer patients

**Fig. 5:**
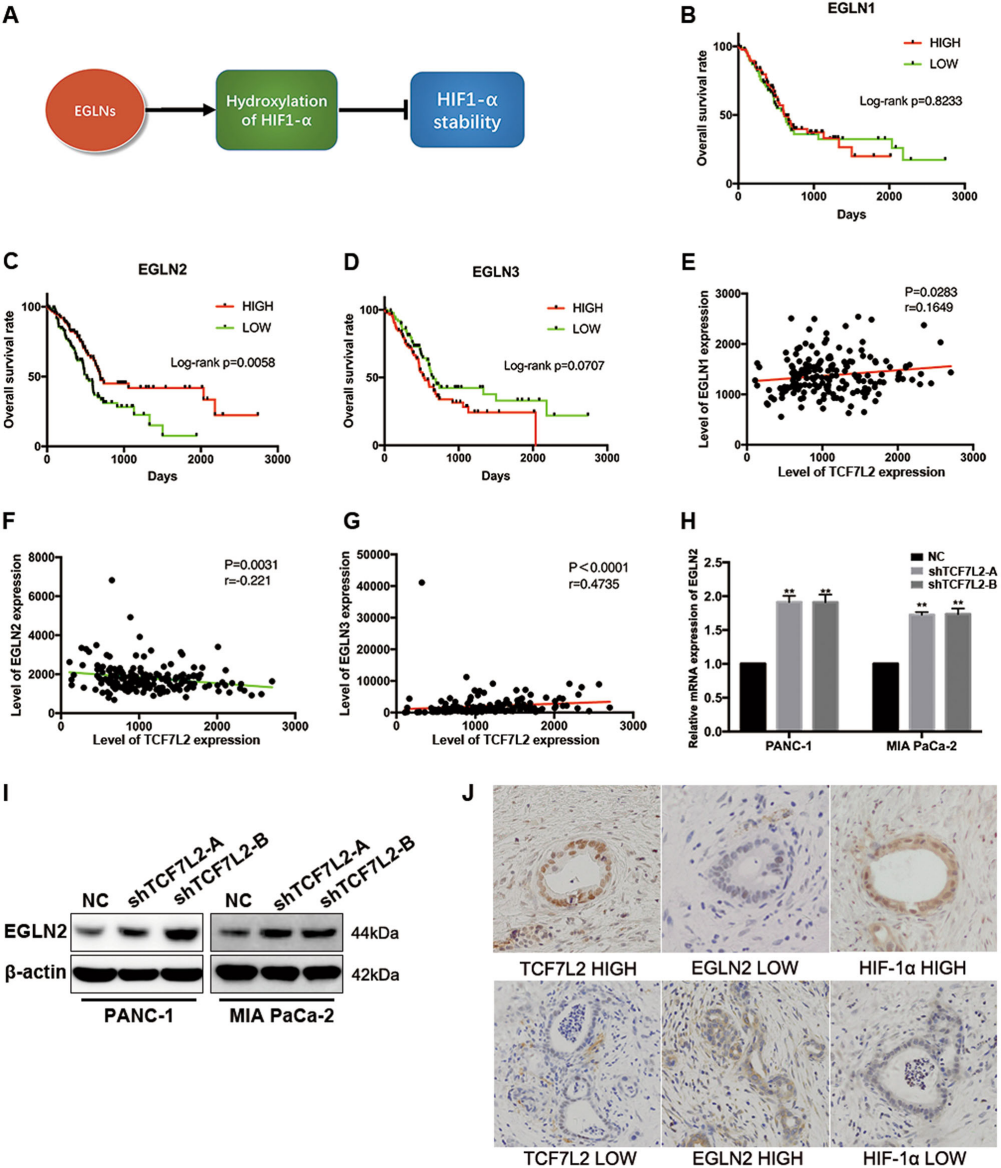
EGLN2 negatively correlates with TCF7L2 expression and is a prognostic indicator in pancreatic cancer patients. **a** EGLNs regulated hydroxylation of HIF-1α to ultimately affect HIF-1α stability. **b**–**d** The prognostic value of EGLNs family members, including EGLN1, EGLN2, and EGLN3, showed that decreased expression of EGLN2 predicted a worse prognosis for pancreatic cancer by analysis of the TCGA dataset. **e**–**g** Correlation analysis of the TCGA dataset indicated that TCF7L2 expression was negatively correlated with EGLN2, and was positively correlated with EGLN1 and EGLN3. **h** TCF7L2 knockdown resulted in an increase of EGLN2 levels in PANC-1 and MIA PaCa-2 cells. **P < 0.01 vs. control group. **i** TCF7L2 silencing increased EGLN2 protein levels. **j** EGLN2 expression was low in patients who displayed high expression of TCF7L2 and HIF-1α

The EGLN family members (including EGLN1, EGLN2, and EGLN3) encode enzymes responsible for a post-translational modification to poise HIF hydroxylases to respond to hypoxia in particular cells or tissue. Thus, we hypothesized that TCF7L2 might impact glycolysis reprogramming by regulating the polyubiquitination and degradation of HIF-1α via adjusting the expression of EGLN family members (Fig. 5a). We analyzed the expression status of EGLN1-3 and its correlation with prognosis in the TCGA database. The results demonstrated that among EGLN family members, only decreased expression of EGLN2 resulted in a worse prognosis for pancreatic cancer patients, while the other two family members were not predictive factors for OS (Fig. 5b–d). Moreover, we performed a correlation analysis in the TCGA dataset to examine whether ELGNs were correlated to TCF7L2 expression. We found that TCF7L2 expression was significantly negatively correlated with EGLN2 expression, while the other two had no clear correlations (Fig. 5e–g). This led us to assume that TCF7L2 might negatively regulate EGLN2 expression in pancreatic cancer. To verify this hypothesis, we examined the expression status of EGLN2 in TCF7L2-silenced PANC-1 and MIA PaCa-2 cells. As shown, EGLN2 mRNA and protein levels increased in TCF7L2-silenced pancreatic cancer cells (Fig. 5h–i). To further verify the in vitro cell line observations, the expression statuses of TCF7L2, EGLN2, and HIF-1α were investigated in parallel by IHC in pancreatic cancer patient tissue samples from FUSCC. As shown, patients with high TCF7L2 expression had low EGLN2 expression and high HIF-1α expression, which made the result from TCGA dataset analysis more convincing (Fig. 5j).

### EGLN2 inhibits proliferation and glycolysis in pancreatic cancer

**Fig. 6:**
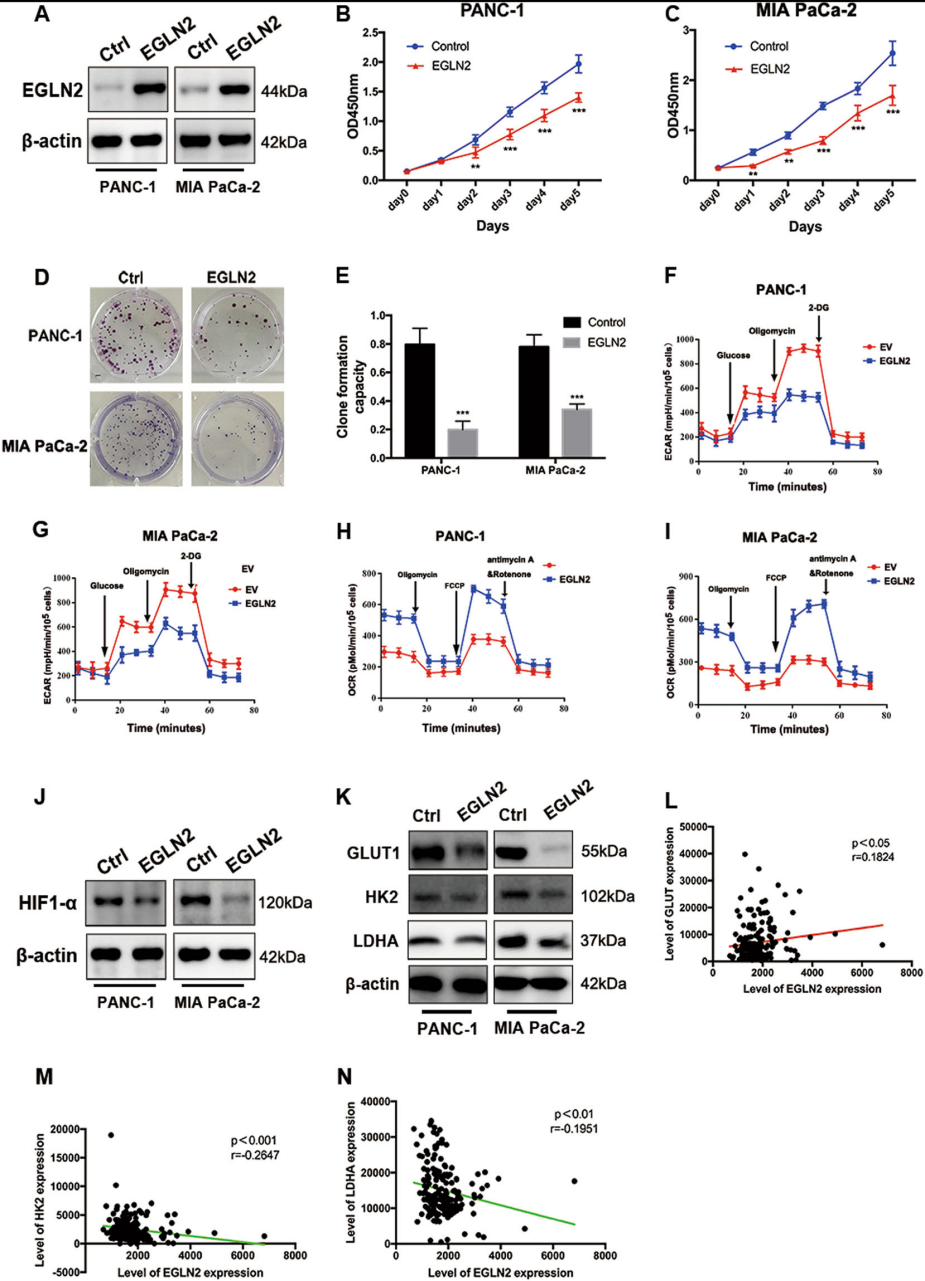
EGLN2 is negatively related to proliferation and glycolysis in pancreatic cancer. **a** The overexpression efficiency of EGLN2 was validated by western blot. **b**, **c** CCK-8 proliferation assays showed that the overexpression of EGLN2 decreased cell viability of PANC-1 and MIA PaCa-2 cells. **d**, **e** EGLN2 overexpression significantly inhibited the clone formation capacity of PANC-1 and MiaPaCa-2 cells. ***P < 0.001 vs. control group. **f**, **g** EGLN2 overexpression decreased glycolytic capacity, as demonstrated by ECAR examination. **h**, **i** EGLN2 overexpression increased OCR values, indicating that EGLN2 is a positive regulator of mitochondrial respiration. **j** EGLN2 overexpression decreased HIF-1α protein levels and **k** relevant glycolytic factors such as GLUT1, HK2, and LDHA in PANC-1 and MIA PaCa-2 cells. **l**–**n** EGLN2 was positively correlated with GLUT1 and negatively correlated with HK2 and LDHA expression, based on TCGA dataset analysis

To explore the function of EGLN2 in PDAC, we overexpressed EGLN2 in PANC-1 and MIA PaCa-2 cell lines. The overexpression efficiency was validated by western blot analysis (Fig. 6a). We then examined the influence of EGLN2 on pancreatic cancer viability and proliferation. As observed, the CCK-8 proliferation assay results demonstrated that overexpression of EGLN2 decreased cell viability maintenance in pancreatic cancer (Fig. 6b, c). Next, we performed a cell cycle analysis. A significant increase in the number of cells arrested in G2/M phase was observed in PANC-1 and MIA PaCa-2 cells when EGLN2 overexpressed, as shown in Supplementary Fig. 1a–d. A subsequent clone formation assay revealed that overexpression of EGLN2 significantly inhibited the clone formation capacity of PANC-1 and MIA PaCa-2 cells (Fig. 6d, e). The impact of EGLN2 on pancreatic cancer glycolysis was assessed by using the Extracellular Flux Analyzer, and showed that overexpression of EGLN2 inhibited glycolytic capacity (Fig. 6f, g). Furthermore, introduction of EGLN2 increased OCR values, indicating that EGLN2 might function as a positive regulator of mitochondrial respiration (Fig. 6h, i). Next, we examined the protein levels of HIF-1α, and, as expected, overexpression of EGLN2 decreased HIF-1α protein levels in PANC-1 and MIA PaCa-2 cells (Fig. 6j). The protein levels of GLUT1, HK2, and LDHA decreased accordingly when EGLN2 was overexpressed (Fig. 6k). Finally, we analyzed the expressional correlation of EGLN2 with GLUT1, HK2, and LDHA in TCGA pancreatic cancer patients. Beyond our expectations, EGLN2 positively correlated with GLUT1 (Fig. 6l). However, inconsistent with our in vitro observations, EGLN2 negatively and significantly correlated with HK2 and LDHA expression in pancreatic cancer patients (Fig. 6m, n). Collectively, these observations supported the conclusion that EGLN2 was a negative regulator of proliferation and aerobic glycolysis in pancreatic cancer.

### EGLN2 is a downstream transcriptional target of TCF7L2 in pancreatic cancer

**Fig. 7:**
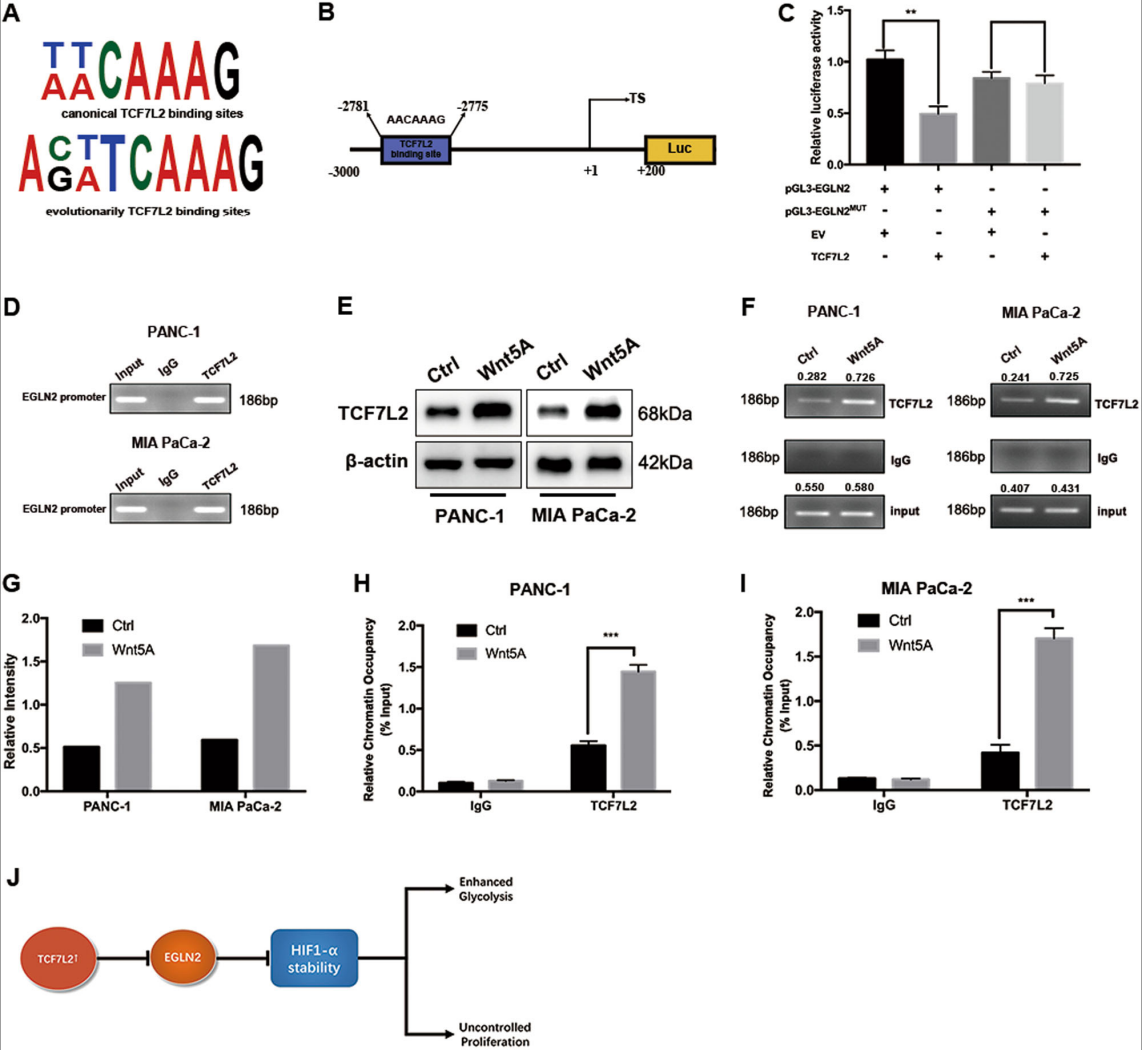
TCF7L2 targets EGLN2 to regulate the HIF-1α axis in pancreatic cancer. **a** Schematic representation of the ELGN2 promoter regions has shown that EGLN2 promoter region contains putative TCF7L2-binding elements. **b** We cloned the promoter region of EGLN2, ranging from −3000 to +200, into pGL3-Basic vector, and observed that TCF7L2 inhibited EGLN2 promoter activity. **c** Dual luciferase assay results suggested that TCF7L2 could suppress EGLN2 promoter activity (**P < 0.01 vs. control group). **d** ChIP assay results with a TCF7L2 antibody demonstrated that TCF7L2 occupied the promoter region, which contained a TCF7L2-binding site. **e** Intracellular TCF7L2 expression increased in PANC-1 and MIA PaCa-2 cells with Wnt5A stimulation. **f-g** Wnt5a treatment increased the occupancy of TCF7L2 on the EGLN2 promoter region. **h-i** TCF7L2 occupied the EGLN2 promoter region when intracellular TCF7L2 protein levels increased. **j** TCF7L2 regulated HIF-1α via EGLN2 to finally affect glycolysis reprogramming

As mentioned above, TCF7L2 positively regulated HIF-1α transcriptional activity and EGLN2 expression negatively correlated with HIF-1α, based on TCGA dataset and FUSCC dataset correlation analysis. This prompted us to question whether EGLN2 was a downstream target of the TCF7L2 transcription factor. TCF7L2 binds preferentially to canonical (T/A-T/A-C-A-A-A-G) and the evolutionarily conserved (A-C/G-T/A-T-C-A-A-A-G) TCF7L2 motifs (Fig. 7a). In the promoter region of EGLN2 from −3000 to +200, there is a putative TCF7L2-binding motif (from −2781 to −2775) (Fig. 7b). We cloned the promoter region from −3000 to +200 into the pGL3-Basic vector. Furthermore, we mutated the TCF7L2-binding motif from AACAAAG to GCCCGAG. Dual luciferase assay results suggested that TCF7L2 could suppress the EGLN2 promoter activity at a ratio of 1:1. However, when the consensus binding site was mutated, TCF7L2 exhibited little impact on the promoter activity (Fig. 7c). Next, we performed a chromatin immunoprecipitation (ChIP) assay, and the results demonstrated that TCF7L2 could occupy the TCF7L2 motifs on the EGLN2 promoter region in PANC-1 and MIA PaCa-2 cells (Fig. 7d). Wnt5a has been reported to activate the β-catenin signalling pathway, which can modulate TCF7L2 expression. To manipulate endogenous TCF7L2 expression, Wnt5a was used to stimulate PANC-1 and MIA PaCa-2 cells and we observed an increase in intracellular TCF7L2 expression (Fig. 7e). Wnt5a treatment increased the occupancy of TCF7L2 on the EGLN2 promoter region (Fig. 7f, g). Finally, we performed a quantitative ChIP assay and further confirmed that TCF7L2 could occupy the EGLN2 promoter region when intracellular TCF7L2 protein levels increased (Fig. 7h, i).

In conclusion, our present study revealed TCF7L2 as a novel predictive marker for prognosis of pancreatic cancer. Mechanist studies demonstrated that TCF7L2 promotes proliferation by maintaining aerobic glycolysis. TCF7L2 suppresses ELGN2 expression, leading to increased HIF-1α protein levels and constitutive transcriptional activation of glycolysis genes (Fig. 7j).

## Discussion

In our present study, we identified TCF7L2 as a novel predictive marker for OS of pancreatic cancer patients, and we provided mechanisms for the metabolism effects, indicating a potential role for TCF7L2 in aerobic glycolysis regulation by targeting the EGLN2/HIF-1α axis.

Metabolic tumour burden has become a new way to evaluate tumour biological behavior based on tumour-specific metabolic reprogramming. Among specific metabolic patterns, glucose metabolism is the most common phenomenon and has been widely measured in the form of ^18^F-FDG PET/CT, which uses visualization technology to display tumour metabolic information as graphics, making it easier for clinicians to understand in clinical work. Our previous studies demonstrated that metabolic tumour burden reflected by PET/CT image technology could be used to predict OS in pancreatic cancer^19^. HIF-1α is a master regulator of hypoxic adaptation and was first discovered by Semenza and Wang in 1992. It regulates various biological processes by controlling various target genes that are related to angiopoiesis, cell survival and apoptosis, amino acid metabolism, nucleosides metabolism, and glycometabolism to maintain cellular environmental homeostasis and adapt to hypoxia and nutrition deficiency conditions. The evidence mentioned above raises conjecture regarding whether TCF7L2 relies on HIF-1α-mediated processes to regulate aerobic glycolysis in PDAC. As mentioned above, TCF7L2 was closely related with several glycolysis genes in common pancreatic cancer cell lines. Aerobic glycolysis has become the basis for the widespread clinical application of ^18^FDG-PET, a practical method reflecting metabolic tumour burden for identifying primary and metastatic tumours. Some previous studies have demonstrated that TCF7L2 played a central role in directing glucose homeostasis, morphology regulation, vascularization, and regeneration in the pancreas. Moreover, TCF7L2 connected mitochondrial biogenesis and metabolic shift with stem cell commitment to hepatic differentiation. However, its impact on cancer cell glycolysis is a phenomenon that has seldom been discussed^20,21^. Thus, it piqued our interest to explore whether TCF7L2 was related to, and regulated expression of, glycolytic genes to affect clinical prognoses in patients with PDAC. The TCGA dataset is a comprehensive and powerful tool to develop a scientific framework used to detect genomic abnormalities involved in cancer and at the same time provides a powerful means to analyze the correlation between different genes and their impact on OS. Our in vitro observations were confirmed by using TCGA dataset analysis in pancreatic cancer patients, and the results from the TCGA cohort were confirmed by using tissue microarrays from the FUSCC cohort. PDAC patients harbour mutations in Kras, p53, Smad4, and CDKN2A; however, the contribution of these genetic mutations to the expression status of TCF7L2 has seldom been discussed. Thus, by using the TCGA dataset analysis and tissues from our own center to analyze the impacts of driver gene mutations on TCF7L2 expression and the underlying molecular mechanisms are needed in the future. These results could promote TCF7L2 as a possible target for treating patients with different driver gene mutations.

TCF7L2 is an important regulator that mediates canonical Wnt/β-catenin signalling in Wnt-activated cells. During Wnt activation, TCF7L2 acts as an activator for Wnt target genes, by which it regulates many Wnt-related biological processes^22–25^. Based on the results in the present study and the impact of the Wnt signalling pathway on the regulation of pancreatic cancer malignancies, we were inspired to question whether Wnt treatment could induce metabolism reprogramming, and further investigation is needed^15^. TCF7L2 has a β-catenin-binding domain and β-catenin has been reported to participate in regulating aerobic glycolysis. Thus, targeting the TCF7L2/β-catenin interaction might be a promising way to cut fuel supply for pancreatic cancer, since cancer cell metabolism has been proven to be a new target for improving cancer cell malignancies^26–30^.

EGLNs are oxygen-sensing enzymes, and can hydroxylate distinct proteins to modulate diverse physiopathological signals. Aberrant regulation of EGLNs results in many human diseases, including cancer. It is well known that EGLNs functions largely depend on the role of HIF-1α in tumours, but the upstream regulatory mechanisms of EGLNs, especially in pancreatic cancer settings, remain unclear^31^. In lung carcinoma cells, overexpression of EGLN2 induces cell cycle arrest and suppresses proliferation via inhibition of the nuclear factor-κB (NF-κB) activity^32^. In human colon and colorectal cancer, overexpression of EGLN2 could inhibit tumour growth^33^. However, the role of ELGN2 in pancreatic cancer has seldom been discussed. Our observations established, for the first time, the negative role of ELGN2 in progression of pancreatic cancer. There are contradictory roles of EGLN2, depending on cell type. For example, EGLN2 has been reported to be an unfavourable predictive factor in breast cancer, but functions as a tumour suppressor in colorectal cancer^34,35^. Colorectal cancer and pancreatic cancer are all gastroenterological cancers and share some similar genetic backgrounds, such as mutations in Kras and SMAD4. Thus, the specific roles of EGLN2 varies depending on cancer types and genetic backgrounds.

Although the implication of EGLN2 in many cancers is accepted, mechanisms accounting for its regulation have seldom been discussed. EGLN2 could regulate HIF-1α by post-translational modification. Furthermore, it could also be transcriptionally regulated by HIF-1β, forming a regulatory loop^36^. In colorectal cancer, decreased ELGN2 expression was not caused by promoter DNA methylation, a mechanism that accounted for gene silencing, indicating that there might be other regulatory mechanisms^37^. In our present study, we identified TCF7L2 as a transcriptional factor that was responsible for EGLN2 silencing, but the in-depth mechanisms remain elusive. TCF7L2 could interact with co-repressors, such as Groucho and histone deacetylase, to silence gene expression^38^. There are other yet unidentified factors, and high-throughput protein–protein interaction screening methods are needed to search for TCF7L2 interacting protein and to further elucidate the underlying mechanisms for EGLN2 silencing in pancreatic cancer.

Collectively, our present study uncovered TCF7L2 and EGLN2 as novel prognostic markers for pancreatic cancer and provided a possible regulatory mechanism. These observations provide directions for further studies of the role of TCF7L2 in pancreatic cancer.

## Methods

### Patients and follow-up

Between January 2010 and December 2012, a total of 118 patients who undergone R0 resection for pancreatic cancer at Shanghai Cancer Center (FUSCC) were recruited to this study. Patients who received radiotherapy, neoadjuvant therapy, and tumor confirmed by postoperative pathology in the less common histologic subtypes of PC such as intraductal papillary mucinous neoplasms or mucinous cystic neoplasms with invasive cancer, neuroendocrine carcinomas, adenosquamous carcinomas, and acinar cell carcinomas were excluded. Definition of margin status is uniform that a margin was considered positive if there were tumor cells at the margin, or within 1 mm of the margin. The finally pathologic assessment was determined by two experienced pathologists (Wensheng Liu and Wenyan Xu) with agreement in all cases. Adjuvant chemotherapy was recommended to each patient in 1 month after surgery though a few patients delayed adjuvant chemotherapy because of physical performance and others. Regimens for adjuvant chemotherapy were generally based on gemcitabine. Informed consent was obtained from each patient according to the Institutional Review Board’s guidelines.

All patients were regularly followed until death to monitor disease progression according to a standardized protocol. Briefly, each patient was assessed 4 weeks after surgery and then at least every 2 months via clinical and laboratory examinations, including serum CA19-9 determination. Image examinations (computed tomography scans, magnetic resonance imaging, bone scans, and positron emission tomography/computed tomography) were performed if recurrence or metastatic disease was suspected. OS was calculated as the time interval between the date of surgery and the date of death or the last follow-up visit.

### TCGA database analysis

TCGA-PAAD on RNA expression (Level 3) of pancreatic cancer patients in terms of RNA-Seq by Expectation-Maximization was downloaded from the Cancer Genomics Brower of the University of California, Santa Cruz (UCSC) (https://genome-cancer.ucsc.edu/). In total, 159 primary pancreatic cancer samples from patients with detailed expression data were chosen from the updated TCGA database according to parameters mentioned.

### Cell culture

The human pancreatic cancer cell lines PANC-1 and MIA PaCa-2 were obtained from ATCC and cultured according to standard ATCC protocols. Briefly, PANC-1 cells were cultured in Dulbecco’s Modified Eagle’s Medium (DMEM), containing fetal bovine serum (FBS) at a final concentration of 10%. MIA PaCa-2 cells were cultured in DMEM medium, with FBS concentration of 10% and horse serum at a concentration of 2.5%.

### Protein extraction and western blot analysis

Cells were washed thrice with ice-cold PBS and lysed in RIPA buffer for 10 min, followed by subsequent centrifugation at 12,000 rpm for 25 min at 4 °C to remove debris. Twenty micrograms total protein lysate was subjected to electrophoresis in denaturing 10% SDS-polyacrylamide gel, and then transferred to a membrane for subsequent blotting with specific antibodies. Antibodies against β-actin (Proteintech, 60008-1-lg), LDHA (Proteintech, 19987-1-AP), HK2 (Proteintech, 22029-1-AP), GLUT1 (Proteintech, 66290-1-lg), and HIF-1α (Proteintech, 20960-1-AP) were manufactured by Proteintech. Antibodies against EGLN2 (Abcam, ab108980) and TCF7L2 (Abcam, ab76151) were manufactured by Abcam.

### RNA isolation and quantitative real-time PCR

Total RNA was prepared using TRIzol reagent (Invitrogen, USA). TaKaRa PrimeScript RT Reagent Kit was used for reverse transcription to obtain cDNA for subsequent quantitative real-time PCR analysis. Expression statuses of designated genes and β-actin were determined by quantitative real-time PCR using an ABI 7900HT Real-Time PCR system (Applied Biosystems, USA). All reactions were run in triplicate. Primers sequences are listed in Supplementary Table 1.

### Lentivirus production and infection

We used lentivirus transfection methods to create cell lines stably expressing shRNA oligos directed against TCF7L2. The pLKO.1 TRC cloning vector (Addgene plasmid 10878) was used to generate shRNA oligos against TCF7L2. 21 bp targets against TCF7L2 were AGAGAAGAGCAAGCGAAATAC and TAGCTGAGTGCACGTTGAAAG. Lentivirus was obtained by co-transfection of pLKO.1-shTCF7L2 constructs with psPAX2 and pMD2.G in a ratio of 4:3:1 into HEK-293T cells. shRNA-expressing pancreatic cancer cell lines were obtained by transfection of lentiviral particles and subsequent puromycin selection.

### Measurement of half-life of HIF-1α protein

PANC-1 and MIA PaCa-2 cells were seeded in 10 cm culture dishes. HIF-1α protein were induced by treating cells with CoCl2 (150 μM) for 8 h. Then, the protein synthesis inhibitor CHX was used to treat cells at a concentration of 20 μg/ml^39^. The proteins were harvested at the indicated time points followed by western blot, and the scale of the bands were analyzed by ImageJ software.

### Analysis of promoter activity with dual luciferase assay

HRE-luciferase construct (Addgene plasmid 26731) containing three hypoxia response elements (24-mers) from the Pgk-1 gene, was obtained from Addgene. Promoters of the human EGLN2, spanning from −3000 to 200 of the transcription starting site, were cloned into pGL3-Basic vector. Cells were plated in 96-well culture plates and transfected with pGL3 constructs and Renilla luciferase expression vectors by using Lipofectamine™ 2000 (Invitrogen). Next, the cells were assayed for both firefly and Renilla luciferase activities using a dual-luciferase system (Promega), as described in the manufacturer’s protocol.

### ChIP assay

ChIP was performed according to the instructions of the Magna ChIP™ A/G Chromatin Immunoprecipitation Kit (Merck Millipore Corporation). The nuclear DNA extracts were amplified using primers that spanned the EGLN2 promoter region containing putative TCF7L2-binding sites. ChIP grade TCF7L2 antibody was purchased from Cell Signaling Technology. Primer sequences were F: 5′-GTGAGCCACTGCGTCCATCCAGAT-3′ and R: 5′-GTCCATACCTTTCTTCTCCCGGTT-3′. Quantitative ChIP was performed according to previously reported methods^40^.

### Extracellular acidification rate and oxygen consumption rate

Cellular glycolytic capacity and mitochondrial function were measured by using the Seahorse Bioscience XF96 Extracellular Flux Analyzer, all according to the manufacturer’s instructions for the Seahorse XF Glycolysis Stress Test Kit and Cell Mito Stress Test Kit.

### Immunohistochemical staining

Immunohistochemistry for TCF7L2 and EGLN2 was performed. Briefly, paraffin sections were baked for 60 min at 70 °C, de-paraffinized in xylene, rehydrated in gradually varied alcohol, and the sections were treated with 3% H_2_O_2_ to neutralize endogenous peroxidase for 30 min. The antigen retrieval was carried out with citrate buffer (pH = 6.0) in a pressure cooker. After antigen retrieval, the sections were incubated with primary antibody and secondary antibody. TCF7L2 antibody (Abcam, ab76151) was used at a dilution of 1:100. EGLN2 antibody (Abcam, ab108980) was used at a dilution of 1:50. The sections were then stained with DAB (3,3-diaminobenzidine) and terminated in PBS, and then counterstained with haematoxylin. Based on the staining intensity of TCF7L2 in each case, the sections were scored as follows: 0, negative; 1, weak; 2, moderate; 3, strong. Two observers graded the score of staining intensity independently.

### Statistical analyses

Statistical analyses were performed by SPSS software (version 20.0, IBM Corp., Armonk, NY, USA) and GraphPad Prism (version 7, GraphPad Software Inc., USA) using independent Student’s *t*-test (two-tailed) or one-way analysis of variance. Spearman correlation analysis and logistic regression were used to determine the correlation between TCF7L2, EGLN1, EGLN2, EGLN3, GLUT1, HK2, and LDHA expression levels and clinicopathological characteristics in the TCGA cohorts. Statistical significance was based on two-sided *P-*values of <0.05.

## Electronic supplementary material

Supplementary Figure 1

Supplementary Table 1

Supplementary Table 2

Supplementary figure legends
