# Supplementary figures and images for: TCF7L2 positively regulates aerobic glycolysis via the EGLN2/HIF-1α axis and indicates prognosis in pancreatic cancer
Source: Cell Death Dis. 2018 Feb 23;9(3):321. doi: 10.1038/s41419-018-0367-6 (PMC5833500; doi:10.1038/s41419-018-0367-6)

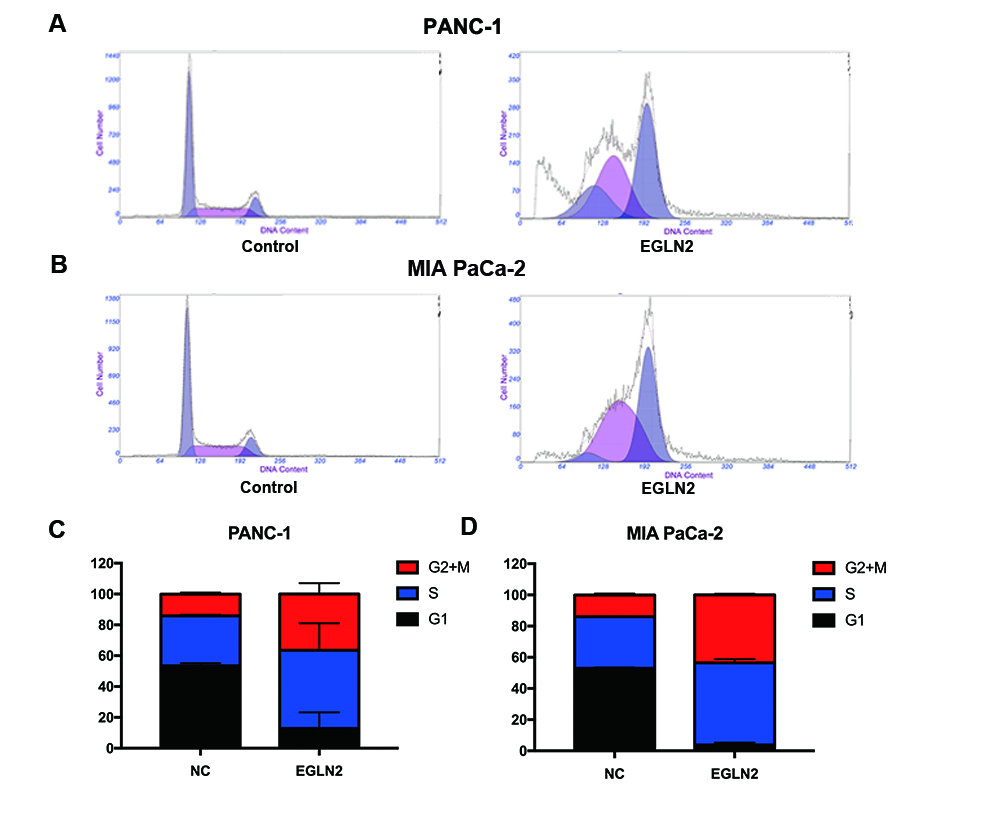

Supplement: Supplementary file 1 — Supplementary Figure 1 [file 41419_2018_367_MOESM1_ESM.tif]
