# Supplementary material for: TCF7L2 positively regulates aerobic glycolysis via the EGLN2/HIF-1α axis and indicates prognosis in pancreatic cancer
Source: Cell Death Dis. 2018 Feb 23;9(3):321. doi: 10.1038/s41419-018-0367-6 (PMC5833500; doi:10.1038/s41419-018-0367-6)
Supplement: Supplementary file 2 — Supplementary Table 1 [file 41419_2018_367_MOESM2_ESM.docx]

**Supplementary Table 1. Primers used in the manuscript**

| **Gene Name** | **Direction** | **Sequence (5'-3')** |
| --- | --- | --- |
| **Glut1** | **Forward** | **CTTTGTGGCCTTCTTTGAAGT** |
|  | **Reverse** | **CCACACAGTTGCTCCACAT** |
| **HK2** | **Forward** | **GATTGTCCGTAACATTCTCATCGA** |
|  | **Reverse** | **CTTGCAGCAGGGCCAGGCAGTCAC** |
| **LDHA** | **Forward** | **TGGAGATTCCAGTGTGCCTGTATGG** |
|  | **Reverse** | **CACCTCATAAGCACTCTCAACCACC** |
| **TCF7L2** | **Forward** | **GACAAGCAGCCGGGAGAGACCAATG** |
|  | **Reverse** | **GGGGGAGGCGAATCTAGTAAGCT** |
| **EGLN2** | **Forward** | **GACCGGTTGCTCATTTTCTGGTC** |
|  | **Reverse** | **TAGGCGGCTGTGATACAGGTACTT** |
| **β-actin** | **Forward** | **CTACGTCGCCCTGGACTTCGAGC** |
|  | **Reverse** | **GATGGAGCCGCCGATCCACACGG** |
