# Supplementary material for: TCF7L2 positively regulates aerobic glycolysis via the EGLN2/HIF-1α axis and indicates prognosis in pancreatic cancer
Source: Cell Death Dis. 2018 Feb 23;9(3):321. doi: 10.1038/s41419-018-0367-6 (PMC5833500; doi:10.1038/s41419-018-0367-6)
Supplement: Supplementary file 3 — Supplementary Table 2 [file 41419_2018_367_MOESM3_ESM.docx]

**Supplementary Table 2.**

Clinicopathological features and correlation of TCF7L2 expression in PDAC in the TCGA cohorts.

| **Supplementary Table 1. Clinicopathological features and correlation of TCF7L2 expression in PDAC in the TCGA cohorts** | | | | | |
| --- | --- | --- | --- | --- | --- |
|  |  | **TCF7L2-Low** | **TCF7L2-High** | **Spearman correlation** | **P value** |
| **Characteristics** | **No.** | **n=79** | **n=80** |  |  |
| Age(y) |  |  |  | 0.004 | 0.958 |
| <60 | 50 | 25 | 29 |  |  |
| ≥60 | 109 | 54 | 55 |  |  |
| Gender |  |  |  | -0.069 | 0.388 |
| Male | 88 | 41 | 47 |  |  |
| Female | 71 | 38 | 33 |  |  |
| Tumor size(cm) |  |  |  | 0.083 | 0.297 |
| ≤3.0 | 58 | 32 | 26 |  |  |
| ＞3.0 | 101 | 47 | 54 |  |  |
| Tumor differentiation |  |  |  | -0.074 | 0.352 |
| Well | 25 | 17 | 8 |  |  |
| Moderate | 86 | 38 | 48 |  |  |
| Poor | 48 | 24 | 24 |  |  |
| Lymph node status(stage) |  |  |  | -0.066 | 0.409 |
| N0 | 46 | 22 | 24 |  |  |
| N1 | 65 | 30 | 35 |  |  |
| N2 | 48 | 27 | 21 |  |  |
| Stage |  |  |  | -0.038 | 0.635 |
| I-IIA | 42 | 26 | 16 |  |  |
| IIB-IV | 117 | 53 | 64 |  |  |

TCF7L2^Low^--negative/weak TCF7L2 expression; TCF7L2^High^--moderate/strong TCF7L2 expression; Lymph node status (stage) was defined by the AJCC 8th edition (N0 = node negative, N1:1 to 3 nodes positive for metastatic disease, N2: more than 4 nodes positive for metastatic disease); P values were derived with Spearman rank correlation coefficient test; all statistical tests are two sided.
