# Supplementary material for: TCF7L2 positively regulates aerobic glycolysis via the EGLN2/HIF-1α axis and indicates prognosis in pancreatic cancer
Source: Cell Death Dis. 2018 Feb 23;9(3):321. doi: 10.1038/s41419-018-0367-6 (PMC5833500; doi:10.1038/s41419-018-0367-6)
Supplement: Supplementary file 4 — Supplementary figure legends [file 41419_2018_367_MOESM4_ESM.docx]

**Supplementary Figure 1 Overexpression of EGLN2 suppresses proliferation in PANC-1 and MIA PaCa-2 cells in vitro**

(a-c) PANC-1 and MIA PaCa-2 cells were examined using PI staining and the cell cycle distribution was measured by flow cytometric analysis. A significant increase in the number of cells arrested in G2/M phase was observed in PANC-1 and MIA PaCa-2 cells when EGLN2 was overexpressed (Overexpression of EGLN2 vs Control, P < 0.01)
